# Editorial: Human Connection as a Treatment for Addiction

**DOI:** 10.3389/fpsyg.2022.964671

**Published:** 2022-07-12

**Authors:** Andrea D. Clements, Human-Friedrich Unterrainer, Christopher C. H. Cook

**Affiliations:** ^1^East Tennessee State University, Johnson City, TN, United States; ^2^Uplift Appalachia, Johnson City, TN, United States; ^3^Strong Brain Institute, East Tennessee State University, Johnson City, TN, United States; ^4^Center for Integrative Addiction Research (CIAR), Grüner Kreis Society, Vienna, Austria; ^5^University Clinic for Psychiatry and Psychotherapeutic Medicine, Medical University Graz, Graz, Austria; ^6^Department of Religious Studies, University of Vienna, Vienna, Austria; ^7^Institute for Medical Humanities, Durham University, Durham, United Kingdom

**Keywords:** addiction, addiction treatment, human connection, multidisciplinary views of addiction, attachment and addiction, religious and spiritual addiction views

The aim of this Research Topic, *Human Connection as a Treatment for Addiction*, is to bring together scholars from various fields to explore the question of whether intentionally increasing meaningful, caring interaction between people may reduce substance and/or non-substance related addictive behaviors. Previous research supports the role that social connection may play in the initiation and maintenance of addiction in both animals and humans (van der Eijk and Uusitalo, 2016; Christie, 2021).

Animal models have shown that neuro-hormonal development, specifically the endogenous opioid and oxytocin systems, is shaped by early experiences possibly explaining the link between early adversity and later substance use patterns (Panksepp, 2004; Machin and Dunbar, 2011; Panksepp and Biven, 2012), and rodents with access to social interaction use fewer substances than those that are isolated (Crofton et al., 2015).

In humans, having a cohesive support/social network and healthy attachments in childhood predict low risk of later addiction (Heilig et al., 2016; Christie, 2021). Treatment and recovery regimens that often foster connection such as 12-step programs and therapeutic communities have shown benefit in reducing substance use (De Leon and Unterrainer, 2020). While having early, close human connection such as maternal/child bonding seems to predict low risk of problematic substance use, lack of such connection often predicts increased risk. Adverse Childhood Experiences (ACEs) including neglect or disruptions in attachment have repeatedly been shown to predict later addiction (Felitti, 2004) and individuals who are addicted to substances are often socially excluded and marginalized, findings which have been supported neurobiologically (Heilig et al., 2016). Individuals decrease pursuit of interpersonal connections and social bonds when they use substances that activate opioid receptors (substances of abuse and treatment medications such as methadone, buprenorphine, and naltrexone) (Inagaki et al., 2015; Torres, 2019; Toubia and Khalife, 2019). Granted, problematic substance use can be initiated or fueled by some types of social interaction, such as affiliation with a substance using social network, thus the investigation of qualitative aspects of human connection is paramount.

With this strong foundation of previous research, a next logical area of research is to investigate whether fostering healthy human connection can actually be used as an intervention or treatment for addiction. Our goal of exploring this question across disciplines was achieved as this issue includes contributions from addiction science, neurobiology, psychology, anthropology, theology, ethics, philosophy, ACEs, science, nursing, psychiatry, criminology, education, chemistry, political science, preventative medicine, and public health. In order to impose structure on this widely varying group of articles, we will group them into three sections according to focus: theoretical, methodological, and empirical.

## Section 1: Theoretical

Wesselmann and Paris pull together previous research on the relationship between trauma and problematic substance use, recommending that “social exclusion” should be considered a form of trauma. They propose that treatment providers consider social exclusion as trauma and attempt to “treat” the social exclusion as a way to reduce substance (mis)use. Addressing social exclusion as a risk factor for substance (mis)use supports the idea that “social inclusion” or increasing human connection as a treatment may have merit.

A much more narrowly focused theoretical piece by Anugu et al. connects the endogenous opioid system with human connection in autism. Anugu et al. propose that one reason individuals on the autism spectrum avoid interpersonal interaction is because they have high opioid tone, meaning that they either overproduce endogenous opioids or need lower levels of opioids to remain at homeostasis. This case study reports on a trial of naltrexone to block the ability of opioids to activate opioid receptors in an autistic patient. Naltrexone treatment was shown to effectively improve the ability to interact interpersonally.

In the remaining four theoretical articles, the role of social connectedness is addressed from the perspective of neuro-psychoanalytic as well as evolutionary psychology. In humans, as in all mammals, social connectedness can be identified as a very strong negative predictor of mental illness in general, and in addictions in particular. All four papers independently refer to the seminal work of Jaak Panksepp and colleagues.

Alcaro et al. give an evolutionary and neurobiological overview of the development and maintenance of addictions. The authors point out that a shortcoming of most neurobiological explanations of addiction leave out the interpersonal/sociocultural aspects. Giacolini et al. link neurobiological research with both primate and human interpersonal interaction, which is also supportive of the idea that lack of healthy connection contributes to addiction. Ringwood et al. pick up the idea that the SEEKING system is hijacked and that MAT keeps it hijacked. Accordingly, interpersonal relationships are discussed as a potential antidote to addictive disorders. Lastly, Mosri emphasizes social connection (to treatment providers and others) as necessary to address the social, psychological, and neurological problems associated with addiction.

## Section 2: Methodological

Of the four methodological articles included in this Research Topic, three involve instrument/app development and three involve collaboration with communities of faith or measurement of some aspect of spirituality. Clements et al. report on an initial validation of an instrument used to assess readiness of church congregations to address addiction. If human connection is found to be a viable treatment for addiction, having individuals willing to make such connections is paramount and the faith community is a potential source of such individuals.

A second article by Clements et al. identifies communication challenges among the faith community, the scientific community, and the clinical community. Communities of faith have long been seen as a potential source of social support for those living with addictions as part of their treatment and recovery. The healthcare community currently manages much addiction treatment, thus fostering communication among these constituencies is important. The authors recommend employing trauma informed principles in health communication. They propose that improving faith/science/clinical collaboration to address addiction could increase the availability of people able to develop meaningful, caring relationships with people living with addictions.

The article by Beck et al. begins with an overview of mutual support groups, emphasizing that they are characterized by interpersonal relationship and are typically quite helpful for those in recovery from problematic use of many types of substances. The authors describe a phone app that can be used to monitor activity and aid in evaluating the effectiveness of a particular non-faith-based mutual support recovery program, Self-Management and Recovery Training (SMART) Recovery. Tools such as this can be valuable as the veracity of a theory of connection as treatment for addiction is tested.

Finally, Fuchshuber and Unterrainer report on the development of a short version of the MI-RSWB 48 (Unterrainer et al., 2010), with the number of items reduced from 48 to 12. Therefore, the MI-RSWB 12 includes four subscales (instead of six in the long version of the scale): Hope, Forgiveness, General Religiousness and Connectedness. The instrument should be particularly useful in clinical settings, for example, to further explore the role of spirituality in the treatment of addiction patients.

## Section 3: Empirical

Our goal in this Research Topic is ultimately to motivate empirical study that will confirm whether or not improving human connection is a viable treatment for addiction. Nine articles are included that empirically test various aspects of this idea. A few of the papers were very closely aligned with the overall theme of this Research Topic and some investigated very targeted topics that support very specific aspects of the theme. The article by Warren et al., reporting findings from a retrospective Therapeutic Community (TC) chart review, said that TCs are an example of an intervention that attempts to create interpersonal connection to reduce substance use. Although a study of a very specific outcome, type and amount of feedback given after receiving feedback, it does give an excellent overview of the TC model in addressing addiction. TC s are an established addiction treatment intervention that supports interpersonal connection as a way to curtail substance use.

Another fairly focused article by Menglu et al. assessed whether Tai Chi improves fitness and cognition in individuals who use methamphetamine. Rather than approaching Tai Chi as a spiritual practice, the authors treated it as exercise. This well done randomized controlled trial found that the control group declined in both fitness and cognition, but both remained stable for the Tai Chi group. Cognitive deficits are a great risk of methamphetamine use, so these findings are an encouraging addition to the treatment literature, though the explicit link to human connection is absent.

Two qualitative studies are included. Snoek et al. concluded, after conducting qualitative interviews with alcohol misusing parents, that the parents would have appreciated having a trusted person intervene in their use. This supports the idea that having close interpersonal relationships could lower drug use. If people who know and care about the substance or alcohol using parent, they may intervene, and the person who is using the substances may feel more apt to listen and thereby curtail use or seek help. Respondents were clear the message should come from a trusted person, implying that a relationship must be developed prior to intervention.

Meulewaeter et al., in a qualitative study of substance-using adults who grew up in a household with parents who were addicted to substances, found several themes. As children, respondents felt lonely, neglected, and stigmatized. Their social connections were influenced by parental addiction. Substances were available in the home and respondents were given great amounts of freedom to go places with friends, many of whom used substances. The parental neglect found aligns with ACEs work (Felitti, 2004) and the attachment insecurity reported by Rübig et al.. Both the lack of supervision and great freedom shows how lack of connection with a caring, guiding adult can lead to problematic substance use.

Yang et al. report by applying structural equation modeling, that perceived social support may increase resilience to perceived stress in addiction patients. Social support could be considered in the future as a potentially beneficial variable for mental health in the treatment of addiction.

Three of the empirical articles directly address the theme of social connection. Best et al. studied 1,313 individuals and found that more human connection and higher quality human connection predicted higher levels of recovery capital and greater growth in recovery capital, thus supporting the idea of human connection as treatment for addiction.

Christie et al. compared non-substance users to substance users who used alcohol, marijuana, methamphetamine, non-prescription opioids, and prescription opioids on social, romantic, and general life satisfaction. Respondents in active addiction reported lower satisfaction across all three variables, but those in recovery from substance use did not differ from non-users. This supports that people can reestablish social connections (social life satisfaction) in recovery.

Finally, two articles examined the relationship between attachment and substance use. Burgkart et al. found that a secure attachment system predicts increased use of appropriate emotion regulation strategies. This finding confirms previous literature that emphasizes the importance of considering the attachment dimension in therapeutic interventions. In contrast, no association with substance use was found in the study. However, it must be said that substance use was very low in this sample. Rübig et al. emphasize the importance of attachment, and suggest that disruptions in attachments may impede treatment effectiveness. They suggest that assessing attachment style prior to therapy initiation is important and that enhancing therapeutic alliance for people with insecure attachment is important. This supports the idea of connection being important in addiction treatment and that having attachment issues could inhibit treatment success.

In summary, the results of our Research Topic of articles point to a significantly positive influence of the feeling of connectedness with people on the treatment of addictive disorders. We have approached the topic very broadly, using a bio-psycho-socio-spiritual model of health and illness to give space to different perspectives. Further work could focus in particular on a more precise characterization of the concept of “human connection” not only in relation to addictive disorders but beyond.

## Author Contributions

All authors listed have made a substantial, direct, and intellectual contribution to the work and approved it for publication.

## Conflict of Interest

The authors declare that the research was conducted in the absence of any commercial or financial relationships that could be construed as a potential conflict of interest.

## Publisher's Note

All claims expressed in this article are solely those of the authors and do not necessarily represent those of their affiliated organizations, or those of the publisher, the editors and the reviewers. Any product that may be evaluated in this article, or claim that may be made by its manufacturer, is not guaranteed or endorsed by the publisher.
